# Lead Exposure, B Vitamins, and Plasma Homocysteine in Men 55 Years of Age and Older: The VA Normative Aging Study

**DOI:** 10.1289/ehp.1306931

**Published:** 2014-06-06

**Authors:** Kelly M. Bakulski, Sung Kyun Park, Marc G. Weisskopf, Katherine L. Tucker, David Sparrow, Avron Spiro, Pantel S. Vokonas, Linda Huiling Nie, Howard Hu, Jennifer Weuve

**Affiliations:** 1 Department of Environmental Health Sciences, and; 2 Department of Epidemiology, School of Public Health, University of Michigan, Ann Arbor, Michigan, USA; 3 Department of Environmental Health, and; 4 Department of Epidemiology, Harvard School of Public Health, Boston, Massachusetts, USA; 5 Department of Clinical Laboratory & Nutritional Sciences, University of Massachusetts Lowell, Lowell, Massachusetts, USA; 6 VA Normative Aging Study, Veterans Affairs Boston Health Care System, Boston, Massachusetts, USA; 7 Department of Medicine, and; 8 Department of Psychiatry, Boston University School of Medicine, Boston, Massachusetts, USA; 9 Department of Epidemiology, School of Public Health, Boston University, Boston, Massachusetts, USA; 10 School of Health Sciences, Purdue University, Fort Wayne, Indiana, USA; 11 Dalla Lana School of Public Health, University of Toronto, Toronto, Ontario, Canada; 12 Department of Internal Medicine, Rush University Medical Center, Chicago, Illinois, USA

## Abstract

Background: Lead (Pb) exposure may influence the plasma concentration of homocysteine, a one-carbon metabolite associated with cardiovascular and neurodegenerative diseases. Little is known about the associations between Pb and homocysteine over time, or the potential influence of dietary factors.

Objectives: We examined the longitudinal association of recent and cumulative Pb exposure with homocysteine concentrations and the potential modifying effect of dietary nutrients involved in one-carbon metabolism.

Methods: In a subcohort of the Veterans Affairs (VA) Normative Aging Study (1,056 men with 2,301 total observations between 1993 and 2011), we used mixed-effects models to estimate differences in repeated measures of total plasma homocysteine across concentrations of Pb in blood and tibia bone, assessing recent and cumulative Pb exposure, respectively. We also assessed effect modification by dietary intake and plasma concentrations of folate, vitamin B_6_, and vitamin B_12_.

Results: An interquartile range (IQR) increment in blood Pb (3 μg/dL) was associated with a 6.3% higher homocysteine concentration (95% CI: 4.8, 7.8%). An IQR increment in tibia bone Pb (14 μg/g) was associated with a 3.7% higher homocysteine (95% CI: 1.6, 5.6%), which was attenuated to 1.5% (95% CI: –0.5, 3.6%) after adjusting for blood Pb. For comparison, a 5-year increase in time from baseline was associated with a 5.7% increase in homocysteine (95% CI: 4.3, 7.1%). The association between blood Pb and homocysteine was significantly stronger among participants with estimated dietary intakes of vitamin B_6_ and folate below (vs. above) the study population medians, which were similar to the U.S. recommended dietary allowance intakes.

Conclusions: Pb exposure was positively associated with plasma homocysteine concentration. This association was stronger among men with below-median dietary intakes of vitamins B_6_ and folate. These findings suggest that increasing intake of folate and B_6_ might reduce Pb-associated increases in homocysteine, a risk factor for cardiovascular disease and neurodegeneration.

Citation: Bakulski KM, Park SK, Weisskopf MG, Tucker KL, Sparrow D, Spiro A III, Vokonas PS, Nie LH, Hu H, Weuve J. 2014. Lead exposure, B vitamins, and plasma homocysteine in men 55 years of age and older: the VA Normative Aging Study. Environ Health Perspect 122:1066–1074; http://dx.doi.org/10.1289/ehp.1306931

## Introduction

Elevated homocysteine is a risk factor shared by neurodegenerative conditions, such as cognitive decline (Haan et al. 2007; Tucker et al. 2005) and Alzheimer’s disease (Seshadri et al. 2002), and cardiovascular diseases (CVDs), such as ischemic heart disease and stroke (Homocysteine Studies Collaboration 2002; Wald et al. 2002). Increasing numbers of older adults are at risk for these conditions because of the unprecedented growth in the population of older adults worldwide. Thus, understanding and intervening in the determinants of elevated homocysteine concentrations may lessen the public health burden of these conditions.

Accumulating epidemiologic and experimental research has demonstrated that exposure to lead (Pb) increases the risk of hypertension (Navas-Acien et al. 2007), and recent research has provided strong evidence that Pb exposure is a risk factor for ischemic heart disease (Jain et al. 2007) and overall cardiovascular morbidity and mortality (Navas-Acien et al. 2007). More limited evidence suggests that exposure to Pb may increase plasma homocysteine concentration. This is an important consideration for the many older adults who have sustained long-term exposures to high levels of Pb earlier in their lives from occupational sources, Pb-based paint, and, especially, widespread combustion of leaded gasoline. Although regulatory actions have dramatically reduced these exposures over the last two decades, past exposures cannot be undone. Moreover, as bone turnover increases with age, Pb that has accrued in bone over decades may reenter the circulation, resulting in reexposure (Hu et al. 2007).

**Figure 1:**
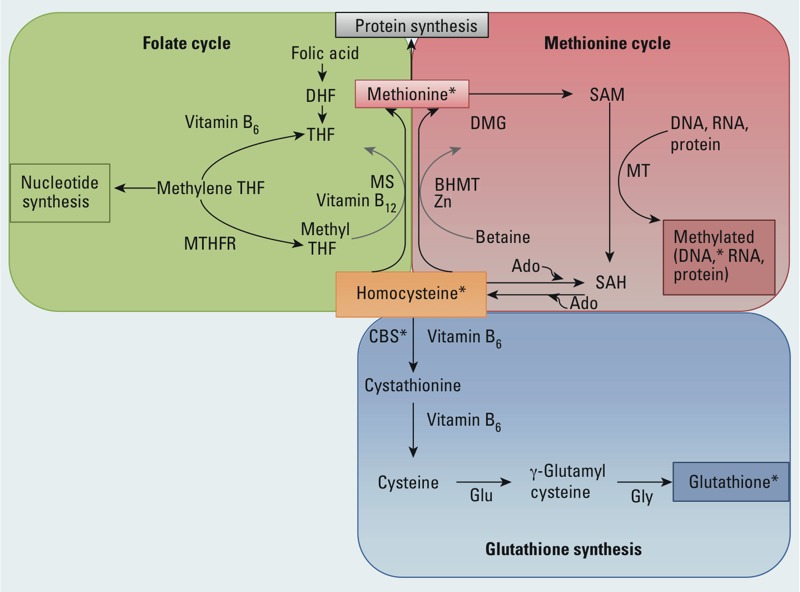
One-carbon metabolism pathway. Homocysteine can be elevated in conditions of low folate, low vitamin B_6_, or low vitamin B_12_. The sulfhydryl groups on several proteins, including cystathionine β-synthase, in the one-carbon metabolism pathway are potential sites for interferences by Pb. Abbreviations: Ado, adenosine; BHMT, betaine-homocysteine *S*-methyltransferase; CBS, cystathionine β-synthase; DHF, dihydrofolate; DMG, dimethylglycine; Glu, glutamate; Gly, glycine; GSH, glutathione; MS, methionine synthase; MT, methyltransferase; MTHFR, methylenetetrahydrofolate reductase; SAH, *S*-adenosylhomocysteine; SAM, *S*-adenosylmethionine; THF, tetrahydrofolate; Zn, zinc. Adapted from Bistulfi et al. (2010). *Indicates putative locations for interference by Pb.

Schafer et al. (2005) proposed that Pb may increase homocysteine concentration by reacting with sulfhydryl groups on several proteins in the homocysteine-processing one-carbon metabolism cycle (Figure 1). Furthermore, in an *in vitro* study, methionine, an amino acid synthesized from the metabolism of homocysteine (in which folate and vitamin B_12_ are cofactors) reduced oxidative stress and related physiological damage in Pb-exposed neuroblastoma cells (Chen et al. 2011). Previous epidemiologic studies have reported higher blood Pb concentration—an indicator of recent exposure to Pb (half-life of Pb in blood is approximately 30 days) (Hu et al. 1998)—in association with higher plasma homocysteine (Chia et al. 2007; Lee et al. 2012; Schafer et al. 2005; Yakub and Iqbal 2010). Little is known, however, about the potential influence of past and cumulative Pb exposures on homocysteine and changes in homocysteine over time. Further, to our knowledge, there have not been any longitudinal studies of the potential influence of the methyl donors—folate and B vitamins—on the association between Pb and plasma homocysteine. If diet can modify the association between Pb exposure and homocysteine, as suggested by findings from an analysis of cross-sectional data from the U.S. National Health and Nutrition Examination Survey (NHANES) (Lee et al. 2012), this could be a critical means for reducing deleterious effects of Pb exposure, even after exogenous exposure has ended.

Therefore, using an established longitudinal study of older men, we evaluated the association between Pb exposure and circulating levels of homocysteine, and the degree to which this association is modified by estimated intakes of methyl-donor nutrients/cofactors (i.e., folic acid, vitamin B_12_, and vitamin B_6_). We estimated the association between recent exposure to Pb, measured by blood Pb concentration, and concurrent homocysteine concentration. We compared associations of recent versus cumulative Pb exposures with homocysteine concentration using concentrations of Pb measured in blood and bone, respectively. Finally, we tested whether associations between Pb exposure and homocysteine were increased among participants whose diets were low in methyl-donor nutrients compared with other men.

## Materials and Methods

*Study population*. From 1961 to 1970, 2,280 men in the greater Boston area, 21–81 years of age, were enrolled in the Veteran’s Affairs (VA) Normative Aging Study (NAS) (Bell et al. 1972). All of the men were free of known chronic medical conditions at enrollment and were invited to participate in health assessments every 3–5 years. Blood Pb measurements were begun in 1977, and bone Pb measurements were begun in 1991. Homocysteine assessment began in 1993. Nearly all homocysteine measurements (> 99%) were made within 30 days of a blood Pb measurement. At each study visit, self-reported smoking status, medication use, physical activity, and dietary intake were assessed.

Of the 1,218 men with blood Pb measurements, 1,056 (99.4%) had at least one homocysteine measurement and data on key covariates (2,301 repeated observations in total). Our analysis of change in blood Pb in relation to change in homocysteine included the 747 men who had at least two repeated measures of blood Pb and concurrent homocysteine (1,830 observations). Of the 851 individuals with bone Pb measurements, 777 (91.3%) had homocysteine and key covariate data (2,158 observations) and were included in our analyses of baseline bone Pb in relation to concurrent and subsequent homocysteine. Loss to follow-up did not vary by baseline Pb level or homocysteine (data not shown).

The human subjects institutional review boards at the Harvard School of Public Health, the Department of Veterans Affairs Healthcare System in Boston, the Brigham and Women’s Hospital (Boston, MA, USA), and the University of Michigan Health Sciences approved the present study. All participants provided written informed consent.

*Assessment of Pb exposure*. We assessed participants’ recent exposure to Pb using concentration of Pb in whole blood, measured via graphite furnace atomic absorption with Zeeman background correction. For the present study, we used blood Pb concentrations from 1993 to 2011 (up to six measures per participant). To assess participants’ cumulative exposure to Pb, we measured baseline Pb concentrations in two bone sites (tibia and patella) via cadmium-109 (Cd^109^) K-shell X-ray fluorescence (KXRF) spectroscopy using previously described methods (Hu et al. 1996). The half-life of Pb in cortical tibia bone is approximately 49 years in this study population (Wilker et al. 2011). Its half-life in trabecular patella bone is shorter, 10–15 years, depending on the study population (Hu et al. 1998).

*Assessment of dietary nutrients*. Annual average diet was assessed with the semiquantitative Willett Food Frequency Questionnaire (FFQ) (van de Rest et al. 2009). The questionnaire assesses usual frequency of consumption of 126 foods (on a scale ranging from never to ≥ 2 times per day) and supplements (no/yes, plus the amount). We estimated participants’ dietary and supplement intakes of three one-carbon metabolism nutrients: folate, vitamin B_6_, and vitamin B_12_. Intakes of vitamins B_6_ and B_12_ were available from all study waves, whereas estimates of folate intake were only available from wave 2 and onward.

*Assessment of plasma homocysteine and B vitamins*. Fasting blood plasma was collected during each clinic visit and frozen at –80°C. Samples were analyzed at the Jean Mayer U.S. Department of Agriculture Human Nutrition Research Center on Aging (HNRCA; Boston, MA, USA). Plasma concentrations of total homocysteine and B vitamins—folate, pyridoxal 5 phosphate (PLP, the activated form of vitamin B_6_), and vitamin B_12_—were measured in fasting blood plasma samples via fluorescence detection with HPLC at the HNRCA (Araki and Sako 1987). Homocysteine detection methods have been previously described (Tucker et al. 2005). The coefficients of variation (CVs) for the biochemical assays of homocysteine, folate, PLP, and vitamin B_12_ were 4.0%, 4.3%, 5.0%, and 4.7%, respectively.

*Statistical analysis*. All analyses were performed using R statistical software (version 2.15.0; R Project for Statistical Computing; http://www.r-project.org/). Means ± SD of Pb markers and geometric means (GMs) [geometric standard deviations (GSDs)] of homocysteine concentrations by key covariates were computed. Tests for linear trend across categories were assessed by assigning integer scores to each ordinal covariate category and modeling as an ordinal variable. We used analysis of variance tests to compare the mean differences of Pb and homocysteine concentrations by smoking status.

We performed cross-sectional analyses of Pb exposure and homocysteine at each individual visit. Using the repeated measures of blood Pb exposure and homocysteine, we estimated the percentage difference in homocysteine per increment in Pb exposure measure using multivariable-adjusted linear mixed-effects models, with random intercepts, of natural log-transformed homocysteine (Fitzmaurice et al. 2004). We analyzed each of the Pb exposure measures separately, modeling repeated blood Pb measurements in relation to concurrent homocysteine, and baseline bone Pb measurements in relation to concurrent and subsequent homocysteine measurements, collectively. We tested for nonlinear trends using generalized additive models with penalized splines in the mgcv R package; because the estimated degrees of freedom for blood and bone Pb concentrations were equal to 1 (i.e., consistent with a linear association between Pb and homocysteine), we used linear models (Hastie and Tibshirani 1990; Wood 2006). All analyses were adjusted for a core set of covariates previously associated with homocysteine: baseline age (years), time since baseline, attained education (< high school, ≥ high school degree), smoking status (current, former, never; time-varying), alcohol consumption (< 2 drinks/day, > 2 drinks/day; time-varying), and body mass index [BMI (in kilograms per square meter); time-varying] (Schafer et al. 2005). We checked for interactions between covariates and time since baseline, but including these terms did not improve the model fit. We further adjusted for intake of methyl-donor nutrients using two approaches. First, we adjusted for continuous plasma concentrations of PLP, vitamin B_12_, and folate concurrent with homocysteine measures. In the second approach, we adjusted for continuous FFQ-based estimates of total energy intake and energy-adjusted dietary intakes (Willett et al. 1997) of vitamin B_6_, vitamin B_12_, and folate as well as supplement use (quantity per day) of vitamin B_6_, vitamin B_12_, and folate, reflecting intake over the previous year. As a sensitivity analysis, we adjusted bone Pb with long-term dietary trend, calculated by averaging FFQ estimates of daily intake over three or more visits for a subset of individuals (*n* = 221). For a secondary test, we restricted blood Pb analyses to individuals who had both plasma and dietary nutrient data. These analyses contained only 1,221 observations from 774 participants. As a sensitivity analysis, we tested to see whether the association between blood Pb and homocysteine was mediated by kidney function (as approximated by serum creatinine and estimated creatinine clearance). Creatinine clearance (in milliliters per minute) was estimated using the equation [(140 – age) × weight (kg)]/[72 × serum creatinine (mg/dL)] (Wu et al. 2003). We compared models of core covariates with blood Pb alone, creatinine alone, and blood Pb and creatinine together.

To explore the possibility that the association of cumulative Pb dose with homocysteine might be mediated through recent exposure (e.g., from Pb released into circulation via bone resorption), we compared results from models of baseline tibia Pb in relation to concurrently assessed homocysteine concentration with and without further adjustment for blood Pb concentration. We performed similar analyses for patella Pb. Although this simple approach for assessing mediation is problematic in many settings, it can generate valid estimates under the following conditions: *a*) both the mediator (in the present study, blood Pb) and outcome (homocysteine) are continuous and can be modeled using linear regression—a condition that was clearly met; *b*) there is no confounding of the mediator–outcome association (e.g., no common causes of homocysteine and bone resorption, which increases blood Pb); and *c*) there is no interaction between exposure and mediator (i.e., at high bone Pb concentrations, the effect per unit increase in blood Pb is no larger or smaller than at low bone Pb concentrations) (Kaufman et al. 2004). As an analysis of the sensitivity of this approach, we also applied a method with a formal basis in causal frameworks (Valeri and Vanderweele 2013) that estimates the total effect of bone Pb on homocysteine along with the indirect effect of bone Pb on homocysteine via blood Pb concentration, and the direct effect of bone Pb on homocysteine via pathways independent of blood Pb concentration (Imai et al. 2010). This procedure, the Baron–Kenny procedure with interaction term, also quantitatively evaluates the impact of interaction (irrespective of magnitude) between the exposure and mediator on the findings.

When participants had at least two study visits, we examined changes in blood Pb concentration over time in relation to concomitant changes in homocysteine. We did this by regressing the visit-to-visit change in homocysteine on change in blood Pb concentration using the general structure, [homocysteine*_t_* – homocysteine*_t –_*_1_] = β_0_ + β_1_[blood Pb concentration*_t_* – blood Pb concentration*_t –_*_1_], where *t* indexes a given visit, *t –* 1 indexes the visit before visit *t*, and the parameter of interest is β_1_, the difference in visit-to-visit changes in homocysteine corresponding to a 1-μg/dL greater visit-to-visit change in blood Pb concentration (e.g., comparing a visit-to-visit increase in blood Pb concentration of 1 μg/dL with no change). For these analyses, we used linear mixed models to account for correlations between repeated dyads of observations from the same individuals (e.g., change from wave 1 to wave 2, change from wave 2 to wave 3). The association between change in blood Pb and change in homocysteine was not linear; therefore, we modeled increases and decreases in blood Pb concentrations using separate terms (reference: no change in blood Pb concentration), effectively a piece-wise regression model with a breakpoint at [blood Pb concentration*_t_* – blood Pb concentration*_t –_*_1_] = 0. We adjusted these analyses for time between visits, age at the start of the interval, and trajectory in smoking status over the interval (i.e., always a former smoker, quit smoking, always a smoker, began smoking, always a never-smoker). Adjusting for additional covariates did not change the findings. These analyses involved the data of 1,245 visit-to-visit intervals from 747 participants.

To examine whether past or cumulative Pb exposure might influence the long-term trajectory of homocysteine concentration with age, we compared trajectories in homocysteine (as percentage change from baseline homocysteine) by tibia bone Pb concentration at baseline. To do so, we amended our mixed-effects linear models for tibia Pb concentration with a cross-product term between time since baseline and tibia Pb concentration. This model also contained a cross-product term between time and baseline age. The inclusion of other time–covariate cross-products did not change the results (data not shown).

To compare the association of Pb exposure with homocysteine by intake of methyl-donor nutrients, we fit models that incorporated all observations and included a cross-product term between each Pb exposure measure and dichotomous nutrient measure (median, or equal to/above vs. below the median). We also compared men who were below the median for all nutrient measures (*n* = 517, plasma; *n* = 383, diet) with men above the median for all nutrient measures (*n* = 473, plasma; *n* = 376, diet). In our primary analyses of the potential interaction of blood Pb concentration with dietary nutrients, we fit models stratified according to intake from food and then further adjusted for supplement use (yes, no), thus treating supplement use as a surrogate for confounding by socioeconomic status (SES), especially in the stratum of high intake. Although dietary supplements make up part of the total nutrient intake, use of these supplements is also related to SES, which predicts Pb exposure, and can affect dietary choices in complex ways (Radimer et al. 2004; Rock 2007). Median estimated dietary intakes of folate and vitamins B_6_ and B_12_ were 343 μg/day, 2.24 mg/day, and 6.19 μg/day, respectively. Median plasma nutrient concentrations were 11.6 ng/mL folate, 64.1 nmol/L B_6_, and 449 pg/mL B_12_.

We also tried multiple alternative analytical approaches. We stratified analyses by supplement use (yes/no) among all participants, by supplement use (equal to/above vs. below the median) restricted to supplement users, and by total intake (dietary and supplement). Analyzing supplement data entails complex considerations. In addition to its patterning by SES, particularly during the years of the present study, supplement use dramatically increased total nutrient intakes (often by an order of magnitude or more), yet trials of supplement use in healthy populations have yielded equivocal findings with respect to several health effects (Fortmann et al. 2013). To circumvent these complexities, we also conducted sensitivity analyses that were stratified by dietary intake, restricted to nonsupplement users.

## Results

Of the 1,056 men in our study, 55–97 years of age at baseline, mean (± SD) concentrations of Pb in blood and tibia bone were 4.9 ± 2.7 μg/dL and 21.4 ± 13.5 μg/g, respectively (Table 1). The mean (± SD) patella Pb concentration was 30.6 ± 20.1 μg/g (data not shown). At baseline, the GM (GSD) homocysteine concentration was 10.1 (1.3) μmol/L. Baseline plasma concentrations of vitamin B_12_ (98–4,081 pg/mL) and folate (1–69 ng/mL) were also similar to those of U.S. men > 60 years of age (Pfeiffer et al. 2005). Baseline plasma PLP (vitamin B_6_) nutrient concentrations ranged from 5 to 995 nmol/L. The U.S. recommended daily allowance (RDA) for folate is 400 μg/day, and 279 participants (62.6%) consumed less than the RDA from estimated diet alone. RDAs of vitamin B_6_ and B_12_ are 1.7 mg/day and 2.4 μg/day, respectively. In our population, 233 (22.1%) consumed less vitamin B_6_, and 47 (4.5%) consumed less vitamin B_12_, than the RDA from estimated diet alone.

**Table 1:** Blood Pb, tibia bone Pb, and plasma homocysteine concentrations, by key baseline participant characteristics among study participants with data on blood Pb and homocysteine concentrations (*n* = 1,056).

Characteristic	*n* (%) or mean ± SD	Blood Pb (μg/dL)		Tibia bone Pb (μg/g)		Plasma homocysteine (μmol/L)	
		Mean ± SD	*p*-Value^*a*^	Mean ± SD	*p*-Value^*a*^	GM (GSD)	*p*-Value^*a*^
Overall value		4.9 ± 2.7		21.4 ± 13.5		10.1 (1.3)
Age (years)	69 ± 7.4
50–65	355 (34)	4.6 ± 2.6	0.03	16.5 ± 9.3	< 0.0001	9.8 (1.4)	< 0.0001
66–71	327 (31)	4.9 ± 2.8		21.8 ± 12.3		9.9 (1.3)
72–97	374 (35)	5 ± 2.8		26 ± 16.3		10.7 (1.3)
Education
< High school	80 (8)	5.8 ± 3	< 0.0001	30.2 ± 18.6	< 0.0001	9.8 (1.3)	0.9
High school	303 (29)	5.2 ± 2.9		24.7 ± 15.9		10.2 (1.4)
Some college	288 (27)	4.8 ± 2.7		20.3 ± 11		10.1 (1.3)
College degree	202 (19)	4.5 ± 2.3		18.9 ± 11.3		9.9 (1.3)
> College degree	183 (17)	4.4 ± 2.8		17 ± 9.4		10.2 (1.3)
Smoking status
Never	296 (28)	4.8 ± 3	0.1	20 ± 13.7	0.002	9.8 (1.3)	0.005
Former	698 (66)	4.8 ± 2.6		22 ± 13.6		10.1 (1.3)
Current	62 (6)	5.8 ± 3.2		22.1 ± 11.5		11.2 (1.3)
Alcohol consumption (drinks/day)
< 2	834 (79)	4.6 ± 2.7	< 0.0001	21.4 ± 13.9	0.5	9.9 (1.3)	< 0.0001
≥ 2	222 (21)	5.7 ± 2.9		21.2 ± 12.1		10.8 (1.4)
BMI (kg/m^2^)	28 ± 3.9
< 25	216 (21)	4.9 ± 2.8	0.5	21.4 ± 11.5	0.3	10.1 (1.3)	0.9
25 to < 30	574 (54)	4.8 ± 2.7		21.8 ± 14.3		10.1 (1.3)
≥ 30	266 (25)	4.8 ± 2.8		20.4 ± 13.3		10.1 (1.3)
Plasma vitamin B_6_ (nmol/L)	87.5 ± 87.2
< 61.8	561 (53.1)	5.2 ± 2.9	< 0.0001	23.0 ± 14.2	< 0.0001	10.8 (1.3)	< 0.0001
≥ 61.8	483 (45.7)	4.4 ± 2.5		19.3 ± 12.4		9.4 (1.3)
Plasma vitamin B_12_ (pg/mL)	466.4 ± 222
< 431	566 (53.6)	5.1 ± 2.8	< 0.0001	21.5 ± 12.0	0.5	10.8 (1.3)	< 0.0001
≥ 431	469 (44.4)	4.5 ± 2.6		21.1 ± 14.9		9.3 (1.3)
Plasma folate (ng/mL)	11 ± 7
< 9.8	621 (58.8)	5.2 ± 2.8	< 0.0001	21.5 ± 12.9	0.1	10.7 (1.3)	< 0.0001
≥ 9.8	405 (38.4)	4.4 ± 2.6		20.9 ± 13.8		9.3 (1.3)
Dietary vitamin B_6_ (mg/day)	2.3 ± 0.9
< 1.8	254 (24)	5.4 ± 2.8	< 0.0001	22.8 ± 14.7	0.2	10.6 (1.3)	0.0002
1.8 to < 2.2	255 (24)	4.9 ± 2.8		21 ± 12.4		10.1 (1.3)
2.2 to < 2.8	246 (23)	4.7 ± 2.7		21 ± 13.5		10.2 (1.3)
≥ 2.8	252 (24)	4.4 ± 2.6		21 ± 13.9		9.6 (1.3)
Dietary vitamin B_12_ (μg/day)	7.9 ± 5.5
< 4.4	252 (24)	5 ± 2.6	0.9	22.1 ± 13.8	0.4	10.4 (1.3)	0.2
4.4 to 6.2	253 (24)	4.7 ± 2.8		20.1 ± 12.8		10.1 (1.3)
6.2 to 9.0	250 (24)	4.8 ± 2.8		20 ± 12.6		9.8 (1.3)
≥ 9.0	252 (24)	5 ± 2.8		23.4 ± 14.9		10.2 (1.3)
Dietary folate (μg/day)^*b*^	374.8 ± 178.1
< 248.5	135 (18)	4.5 ± 2.8	0.001	21.7 ± 11.7	0.2	9.9 (1.3)	0.7
248.5 to 327.3	134 (18)	4.0 ± 2.4		21.1 ± 13.1		10.3 (1.3)
327.3 to 440	134 (18)	4.0 ± 2.1		19.3 ± 19.3		10.1 (1.4)
≥ 440	134 (18)	3.6 ± 1.7		19.6 ± 19.6		9.9 (1.3)
^***a***^Calculated using a test for linear trend across ordinal categories of nonmissing data. ^***b***^Calculated at visit 2, due to missing data at visit 1.							

At baseline, homocysteine was positively correlated with blood Pb, tibia bone Pb, and patella bone Pb concentrations (respectively, Pearson’s *r* = 0.27, 0.15, and 0.15; all *p* < 0.001). Pb concentrations were lower among men who were younger and had more education, higher plasma concentrations of the three nutrients, and higher dietary intakes of vitamins B_6_ and folate (Table 1).

*Association of recent Pb exposure with homocysteine*. Recent exposure to Pb, measured by blood Pb concentration, was significantly associated with concurrent plasma homocysteine concentration (Table 2). After adjusting for baseline age, time since baseline, education, alcohol, smoking, and BMI, an interquartile range (IQR) increment in blood Pb (3 μg/dL) was associated with 6.3% higher homocysteine (95% CI: 4.8, 7.8%). This association diminished slightly with further adjustment for plasma concentrations of the three methyl-donor nutrients (5.5% higher; 95% CI: 4.0, 6.9%), but not with further adjustment for dietary intakes of these nutrients plus total energy consumption (6.5%; 95% CI: 4.3, 8.8%). These associations may have varied at least in part because they were based on different subsets of observations due to missing nutrient exposure data. When we restricted the blood Pb analyses to individuals with both plasma and dietary nutrient data, the estimated association between blood Pb concentration and homocysteine varied by < 10% across the core model (6.4%; 95% CI: 4.3, 8.5%), plasma model (5.8%; 95% CI: 3.7, 7.9%), and dietary model (5.9%; 95% CI: 3.8, 8.1%). For comparison, results from the model that included terms for the core covariates and current blood Pb concentration indicated that homocysteine concentrations were 5.7% higher, on average, in association with a 5-year passage of time since baseline (95% CI: 4.3, 7.1%), and 13% higher (95% CI: –0.2, 28.1%) in current smokers compared with never-smokers.

**Table 2:** Adjusted percentage difference (95% CI) in plasma homocysteine per IQR increment in Pb exposure biomarker.

Estimate	Pb concentration		
	Blood (IQR = 3 μg/dL)	Tibia (IQR = 14 μg/g)	Patella (IQR = 21 μg/g)
Core model^*a*^
Observations (*n*)	1,056 (2,301)	777 (2,158)	770 (2,133)
Percentage difference in Hcy per IQR biomarker (95% CI)	6.3 (4.8, 7.8)	3.7 (1.7, 5.6)	3.0 (1.0, 5.0)
*p*-Value	< 0.0001	0.0002	0.003
Core model plus plasma nutrients^*b*^
Observations (*n*)	1,033 (2,240)	767 (2,106)	760 (2,082)
Percentage difference in Hcy per IQR biomarker (95% CI)	5.5 (4.0, 6.9)	3.6 (1.7, 5.5)	3.0 (1.0, 5.0)
*p*-Value	< 0.0001	0.0002	0.003
Core model plus dietary and supplement nutrients^*c*^
Observations (*n*)	779 (1,241)	634 (1,328)	627 (1,314)
Percentage difference in Hcy per IQR biomarker (95% CI)	6.5 (4.3, 8.8)	3.0 (0.8, 5.2)	2.1 (–0.2, 4.4)
*p*-Value	< 0.0001	0.008	0.07
Hcy, homocysteine. ^***a***^Core covariates were baseline age, time since baseline, education, smoking status, alcohol consumption, and BMI; mixed-effects models used random intercepts and unstructured correlation structures. ^***b***^Analyses were further adjusted for plasma nutrients including core covariates and plasma PLP (B_6_), B_12_, and folate. ^***c***^Analyses were further adjusted for dietary nutrients including core covariates, total energy, and total energy–adjusted dietary intakes of vitamin B_6_, vitamin B_12_, and folate as well as supplement use (yes/no) of vitamin B_6_, vitamin B_12_, and folate, based on FFQ responses.			

In additional sensitivity analyses that were further adjusted for creatinine clearance (an intermediate marker of kidney function), the association of an IQR increment in blood Pb concentration with higher homocysteine remained significant (6.3%; 95% CI: 4.8, 7.8%).

*Association of cumulative Pb exposure with homocysteine*. Higher cumulative exposure to Pb, measured by baseline tibia bone Pb concentration, was also significantly associated with elevated plasma homocysteine (Table 2). After adjusting for the core group of covariates, an IQR increment in tibia bone Pb concentration (14 μg/g) was associated with 3.7% higher homocysteine (95% CI: 1.7, 5.6%). Further adjustment for plasma and dietary methyl-donor nutrients did not alter this finding. The corresponding associations with homocysteine per IQR increase in baseline patella Pb concentration (21 μg/g) were consistent with those estimated per IQR increase in tibia Pb concentration (Table 2). For example, from the core covariate-adjusted model, homocysteine concentrations were 3.0% higher per IQR increment in patella Pb concentration (95% CI: 1.0, 5.0).

The association between an IQR increase in tibia Pb at baseline (14 μg/g) and homocysteine (at baseline and follow-up) was substantially attenuated after adjustment for concurrent blood Pb (1.5% higher; 95% CI: –0.5, 3.6%), whereas there was little change in the association between an IQR increase in concurrent blood Pb and homocysteine (5.8% higher; 95% CI: 4.0, 7.6). These models were adjusted for the core covariates (time since baseline, baseline age, education level, smoking status, and BMI). Similarly, the association pertaining to patella Pb was attenuated, but the association pertaining to concurrent blood Pb changed very little when both were included in the same model. These findings are consistent with the results generated by the Baron–Kenny mediation procedure, in which the estimated effect of bone Pb (per IQR increment) on homocysteine mediated through blood Pb (i.e., the average causal mediation effect via blood Pb) was a 2.9% increase in homocysteine (95% CI: 2.0, 4.0); the average direct effect of bone Pb (per IQR increment) on homocysteine via pathways other than blood Pb was 1.7% (95% CI: –0.6, 4.3); and the estimated fraction of the total effect of bone Pb on homocysteine mediated by blood Pb was 0.63. Interaction between bone and blood Pb did not alter these findings.

*Longitudinal changes in blood Pb in relation to changes in homocysteine*. Similar to findings in the U.S. population during the study period, blood Pb concentration among study participants decreased over time, by 0.7 μg/dL, on average, per 3.5-year interval (range, –14 to 8 μg/dL). Among individuals with at least two visits (*n* = 747; 1,245 observations), the average time interval between visits was 3.9 years. Although homocysteine concentration generally increased over time, the degree of change in homocysteine generally tracked changes in blood Pb concentration. In particular, during intervals corresponding to the reference condition (i.e., never smoked, 67.5 years of age at the start of the interval, no change in blood Pb concentration, 1-year interval), homocysteine increased by 0.19 μmol/L, on average. By comparison, visit-to-visit intervals in which blood Pb concentrations declined corresponded to concomitantly smaller increases (or steeper declines) in homocysteine (*p* = 0.02). For each additional 1-μg/dL decline in blood Pb between visits, homocysteine concentration increased by 0.13 μmol/L less (95% CI: –0.25, –0.01). Although visit-to-visit increases in blood Pb concentration were associated with greater increases in homocysteine (0.07 μmol/L greater increase in homocysteine per each additional 1-μg/dL increase in blood Pb), this result was not statistically significant (95% CI: –0.11, 0.25).

*Baseline tibia Pb in relation to change in homocysteine*. Although cumulative exposure to Pb, as measured by baseline tibia bone Pb concentration, was associated with higher plasma homocysteine over the course of follow-up (as reported above), it was not associated with the degree to which plasma homocysteine changed over time, as estimated by the cross-product term between tibia Pb and time since baseline (*p*_time–bone Pb interaction_ = 0.9). Specifically, the estimated percentage difference in homocysteine per IQR increase in baseline tibia Pb (14 μg/g) was statistically consistent across visits: visit 1, 3.6% higher (95% CI: 1.4, 6.0); visit 2, 3.8% higher (95% CI: 1.2, 6.4); visit 3, 4.6% higher (95% CI: 1.7, 7.5); and visit 4, 5.5% higher (95% CI: 1.3, 9.9).

**Figure 2:**
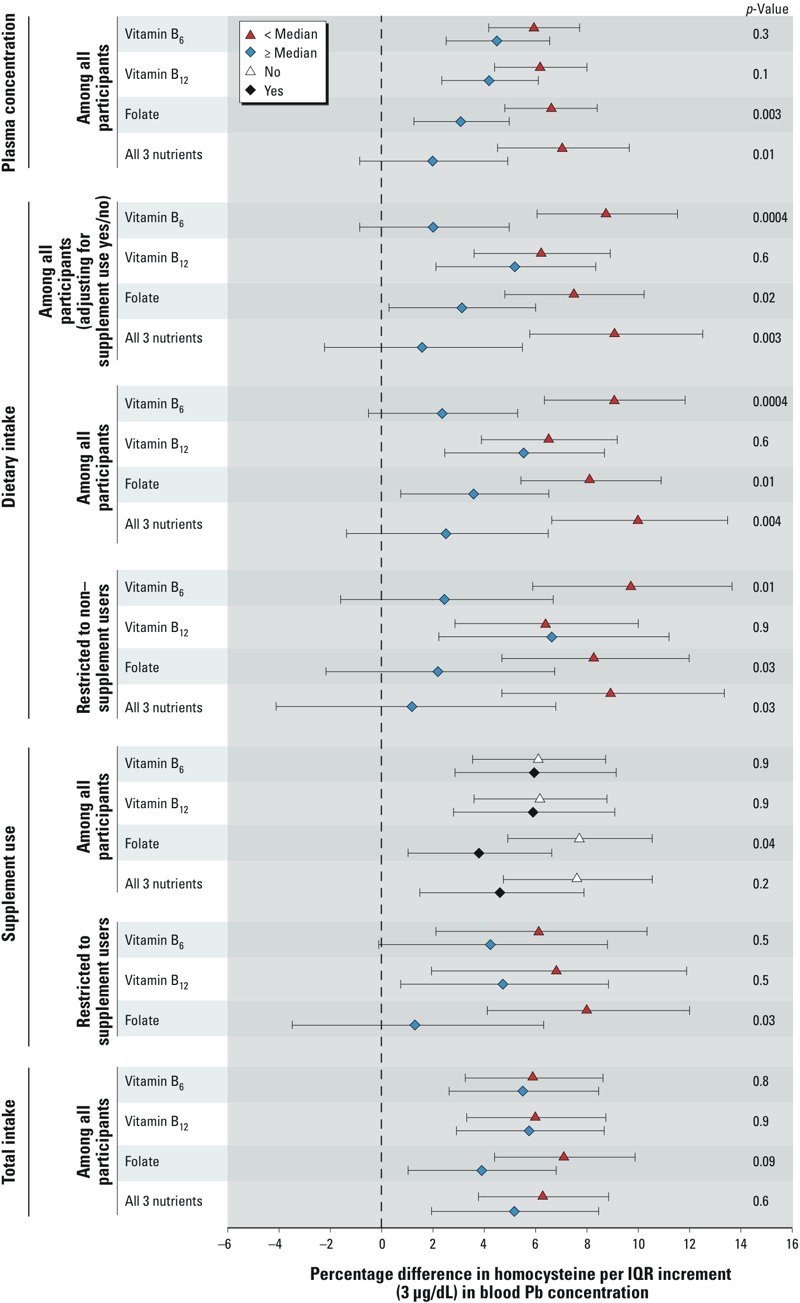
Adjusted percentage difference in homocysteine per IQR increment in blood Pb concentration by nutrient status level. All analyses were adjusted for age, education, alcohol consumption, smoking status, and BMI.

*Association of blood Pb concentration with homocysteine, by intake of methyl-donor nutrients*. The association between blood Pb concentration and concurrent plasma homocysteine was significantly stronger among men whose dietary intakes of vitamin B_6_, folate, or vitamins B_6_, B_12_, and folate (combined) were below the median, even after adjusting for supplement use (Figure 2). For example, among men with lower vitamin B_6_ intake, an IQR increment in blood Pb level was associated with 8.7% higher homocysteine concentration (95% CI: 6.0, 11.5%), compared with 2.0% higher (95% CI: –0.8, 5.0%) among men with higher vitamin B_6_ intake (*p*_interaction_ = 0.0004). Associations between blood Pb and homocysteine also were significantly stronger among men with lower dietary intakes of folate or of all three dietary nutrients combined compared with men whose estimated intakes were equal to or above the median (*p*_interaction_ = 0.02 and 0.003, respectively); and among men with lower plasma folate, or men with lower plasma concentrations of all three plasma nutrients combined, compared with men whose plasma concentrations were equal to or above the median (*p*_interaction_ = 0.002 and 0.006, respectively). In addition, a pattern in which low folate intake conferred greater Pb-related homocysteine risk was present in the results of sensitivity analyses that *a*) incorporated supplement intake into the total intake, *b*) was restricted to supplement users, and *c*) compared users versus nonusers of methyl-donor nutrient supplements (Figure 2). In these analyses, findings on other nutrients were less remarkable. Secondary models were restricted to dietary intake of nonsupplement users *a*) to reduce confounding by supplement use and its correlates; *b*) to reduce error in measurement of total intake; and *c*) to limit the range of intakes to that which can be achieved via diet, especially in light of evidence-based scrutiny directed toward supplement use. We found evidence suggesting that the association between bone Pb and homocysteine varied by plasma and long-term dietary intake of vitamin B_12_ (*p*_interaction-plasma_ = 0.00002; *p*_interaction-diet_ = 0.1), but results were less suggestive with respect to folate (*p*_interaction-plasma_ = 0.1; *p*_interaction-diet_ = 0.8) or vitamin B_6_ (*p*_interaction-plasma_ = 0.5; *p*_interaction-diet_ = 0.1).

## Discussion

In this study of older men with community-level exposure to Pb and typical dietary intakes, recent exposure to Pb, measured by blood Pb concentration, was positively associated with concurrent plasma homocysteine concentration. This association was stronger among men with below-median dietary intakes of vitamins B_6_ and folate, and among men with below-median plasma folate. Homocysteine concentration among men in our study was comparable to that among similarly aged men in the general population (Jacques et al. 1999). The present study contains one of the richest available data collections on Pb exposure, diet, and homocysteine. It is strengthened by its use of both dietary and plasma measures of nutrient intake, and its repeated measures of homocysteine and blood Pb concentrations, in addition to its measures of cumulative Pb exposure, allowing detailed examination of differences and longitudinal changes in homocysteine with recent and cumulative Pb exposure.

In light of other homocysteine predictors, the magnitude of the association of Pb exposure with homocysteine is substantial. On average, homocysteine concentration was 6.3% higher per IQR (3 μg/dL) increment in blood Pb concentration, equivalent to the estimated increase in homocysteine over 5.5 years of follow-up (i.e., 5.5 years of aging), and about half of the estimated difference in homocysteine between current and never-smokers. In the Framingham Heart Study, a 25% increase in plasma homocysteine was associated with a 14% increase in the relative risk of incident Alzheimer’s disease over 8 years of follow-up (95% CI: 6, 22%) (Seshadri et al. 2002), and a meta-analysis of 30 retrospective or prospective studies estimated that a 25% increase in homocysteine (approximately 3 μmol/L) was associated with 11% increase in relative risk of ischemic heart disease (95% CI: 4, 17%) and a 19% increase in relative risk of stroke (95% CI: 5, 31%) (Homocysteine Studies Collaboration 2002). Overall, these findings suggest that, if the association between Pb and homocysteine is causal, a modest population increase in homocysteine due to Pb exposure could dramatically increase the prevalence of cardiovascular and neurodegenerative diseases. Particularly in older adults, where bone turnover is relatively high and current exogenous exposure is generally low, the source of blood Pb may be bone resorption, which liberates historic Pb exposure.

Intakes of methyl-donor nutrients below the median for the study population appeared to strengthen the association between blood Pb and homocysteine. Among men with lower folate dietary intake, the estimated increase in homocysteine associated with an IQR increment of blood Pb was similar to the estimated increase in homocysteine over 9 years of follow-up, whereas among men with higher folate intake, the estimated increase was comparable to that associated with about 3 years of follow-up. We observed this disparity in blood Pb–homocysteine association by dietary nutrient intake, even after adjusting for supplement use or restricting to supplement nonusers. These results are consistent with the possibility that the additional nutrients found in foods may allow vitamins to work optimally (Lichtenstein and Russell 2005), although we could not test this in our data. Thus, preventing Pb exposure may not only reduce the risk of elevated homocysteine, but among those who have been exposed, higher consumption of methyl-donor nutrients may partially mitigate the influence of Pb on homocysteine.

Higher cumulative exposure to Pb, measured by concentrations in bone, also was associated with elevated homocysteine, but this association appeared to be attributable, in large part, to recent Pb exposure. The problems specific to the analysis of discrete outcomes and/or mediators are not present in these analyses (Kaufman et al. 2004); assuming no interaction and unmeasured confounding of the estimated blood Pb–homocysteine association (Cole and Hernan 2002; Kaufman et al. 2004), the results suggest that the influence of Pb exposure on homocysteine is fairly immediate and, thus, that recent exposure to Pb (measured in blood) may drive the association between Pb and homocysteine. However, even with substantial cumulative exposure, interventions that minimize acute elevations in blood Pb concentration (e.g., from endogenous sources) may be an effective strategy to mitigate increases in homocysteine, although there is insufficient information to recommend such an intervention for this purpose at this time. Results from homocysteine-lowering dietary intervention trials among patients with prior disease are mixed: The interventions effectively reduce circulating homocysteine but perform inconsistently in affecting homocysteine-related health outcomes (Mei et al. 2010; Morris 2012). Among the explanations for these inconsistent findings are that *a*) the study populations differed in their preintervention nutritional deficiencies, whereby supplementing deficiencies may help, but supplementing sufficient intake may have no effect or may cause harm (Morris 2012); *b*) the interventions may have begun too late to influence the health trajectory; or *c*) the observational associations of homocysteine with health outcomes may not be causal. Our study is among the first to evaluate whether the association between Pb exposure and homocysteine might be influenced by intake of folate and other B vitamins. In data from NHANES, the association between blood Pb concentration and homocysteine was more pronounced among people with below-median plasma concentrations of vitamin B_6_ or folate than among people with higher plasma concentrations (*p*_interaction_ = 0.002 and 0.06, respectively). However, results for plasma vitamin B_12_ followed the opposite pattern, with a significantly stronger adverse association between blood Pb and homocysteine among those with higher vitamin B_12_ concentration (*p*_interaction_ = 0.01) (Lee et al. 2012). In contrast, although our findings on vitamin B_6_ and folate were consistent with those from the NHANES study, our analyses of vitamin B_12_, whether based on reported intake or plasma concentration, yielded stronger associations between blood Pb and homocysteine among those with lower vitamin B_12_ than among those with higher intakes, but these interactions were not statistically significant.

Our finding that Pb exposure is associated with higher homocysteine concentration is consistent with previous epidemiological studies. Among workers occupationally exposed to Pb in Vietnam, blood Pb concentration and homocysteine were associated, although these analyses were not adjusted for any other factors (Chia et al. 2007). In a cross-sectional analysis of Baltimore Memory Study data from men (mean age, 59 years) with blood Pb and homocysteine concentrations comparable to those in our participants, a 1.0-μg/dL increment in blood Pb was associated with a 0.43-μmol/L increase in homocysteine (Schafer et al. 2005). However, the investigators reported that there was no association between homocysteine and tibia Pb. By comparison, in the present study, a 1.0-μg/dL increment in blood Pb concentration was associated with a 0.30-μmol/L increase in homocysteine (95% CI: 0.23, 0.37). In NHANES participants (mean age, 50 years), homocysteine was higher across progressively increasing quartiles of blood Pb concentration, even though 95% of individuals had blood Pb concentration < 5 μg/dL (Lee et al. 2012). In our study, 55% of participants had blood Pb concentration < 5 μg/dL at baseline. Among adults in Pakistan (18–60 years of age) with higher Pb concentration (mean blood Pb, 11.7 μg/dL), an IQR increase in blood Pb (8.3 μg/dL) was associated with 4.6% higher homocysteine (95% CI: 2.6, 4.8%) (Yakub and Iqbal 2010). The association in the present study was larger: An IQR (3 μg/dL) increase in blood Pb corresponded to 6.3% higher homocysteine.

Our study has several limitations. Errors in the measurement of Pb exposures and homocysteine may have biased and/or reduced the precision of the associations we observed between the two. For example, the time of day of blood draw for homocysteine measurement was not standardized, but homocysteine concentration follows a daily rhythm characterized by an evening peak and nighttime low (Bonsch et al. 2007). In addition, plasma measures of homocysteine assess the pool of homocysteine released after reduction of all disulfide bonds in the sample. Total homocysteine does not include homocysteine thiolactone (a product of misincorporation of homocysteine into proteins and subsequent error-editing) or homocysteine bound to protein by an amide bond (Perla-Kajan et al. 2007). These homocysteine groups are potentially toxic but might not be correlated with plasma homocysteine concentration (Perla-Kajan et al. 2007).

It is possible that our observations reflect effects of Pb on kidney function. Lower kidney glomerular filtration rate (GFR) has been shown to increase homocysteine concentration in a prospective study of community-dwelling adults > 70 years of age (Lewerin et al. 2007). Chronic Pb exposure appears to contribute to chronic kidney disease, indicated by low GFR (Batuman et al. 1983; Brewster and Perazella 2004; Muntner et al. 2003). In the NAS, for example, creatinine clearance was lower with higher long-term exposure to Pb, estimated via patella Pb concentration (Wu et al. 2003). Therefore, Pb exposure might influence homocysteine through an effect on renal function. We performed sensitivity analyses of blood Pb and homocysteine by further adjusting for creatinine clearance as a marker of kidney function, and as an intermediate in the above mechanism. Although the association of blood Pb with homocysteine modestly decreased in magnitude (from a 6.3% increase in homocysteine per IQR increment in blood Pb, to a 5.5% increase), it remained positive and significant, indicating that Pb may elevate homocysteine independently of its renal effects. However, inferences about causal mechanisms should be interpreted with caution.

Some of our analyses had limited statistical power. Missing nutrient data meant that sample sizes were smaller for analyses of Pb exposure, diet, and homocysteine. The change–change analyses also had limited power because participants were lost to follow-up over time. Finally, generalizability of our study may be limited because the study population was older, non-Hispanic white men.

## Conclusions

We found a significant positive association between blood Pb concentration and plasma homocysteine in a cohort of older men. This association between blood Pb and homocysteine was stronger than associations corresponding to baseline bone Pb, and declines in blood Pb concentration over time corresponded with smaller increases (or larger declines) in homocysteine over the same interval, suggesting that circulating Pb may influence circulating homocysteine through Pb’s influence on homocysteine metabolism, even at very low levels of Pb exposure. Our finding that the association between blood Pb and homocysteine was stronger among men with lower estimated intakes of methyl-donor nutrients—especially folate and vitamin B_6_—suggests that nutrient intakes may influence susceptibility to the effects of Pb that might operate through effects on homocysteine. The associations of elevated homocysteine concentration with cardiovascular and neurodegenerative diseases—along with the high prevalence of cumulative and now endogenous exposures to Pb in the population—suggest the potential public health value in researching joint interventions on diet and exposure to Pb.
